# The 1947 Smallpox Vaccination Campaign in New York City, Revisited

**DOI:** 10.3201/eid1005.030973

**Published:** 2004-05

**Authors:** Kent A. Sepkowitz

**Affiliations:** * Infectious Disease Service, New York, New York, USA

**To the Editor:** In 1947, millions of New Yorkers received smallpox vaccinations, an accomplishment still appropriately held up as an example of public health planing and mobilization. Although now mythological, a review of the events of April 1947, from copies of The New York Times (*1*,*2*), tells of a more recognizably human response: pushing, jawing, deceit, shortages, surpluses, and perhaps a unusual way of counting vaccinees.

In March 1947, a patient who had recently visited Mexico traveled by bus to New York City. He became ill, was hospitalized, and, after his death, found to have had smallpox. The occasional case of smallpox had been seen in the area for decades since the last big outbreak in 1875, which had killed 2,000 New Yorkers. However, in 1947, a second case and then a third appeared and concern gathered. On April 4, Israel Weinstein, the New York City Health Commissioner, urged all New Yorkers who had not been vaccinated since childhood to receive another vaccination.

The program worked at the outset. Free vaccine clinics were established throughout the city, and doses were given to private physicians for administration. During the first week, surprisingly little public attention was captured (Times articles typically were brief and confined to page 21). The story hit page 1 on April 13 (*3*), after a second person died from the disease. Mayor William O’Dwyer urged all 7.8 million New York residents to receive the vaccine, and he rolled up his sleeve and was vaccinated by Dr. Weinstein. The city swiftly swung into full crisis mode. Police, fire, and health departments, and hospitals were mobilized to provide additional space for the effort.

Two days later, epidemiologic investigation indicated that all patients with diagnosed cases were related and that, in all likelihood, the outbreak had been successfully halted through tracing the movements of the various patients and vaccinating anyone who had contact with them, so-called “ring” vaccination (*4*). Despite this halt of the outbreak, the city pushed forward. The campaign to “Be sure, be safe, get vaccinated!” had proven successful. By city estimate, >600,000 persons had received vaccine in the first week.

Vaccine side effects, which dominate coverage of today’s vaccination program, were seldom discussed in 1947. Dr. Weinstein assured residents, “Vaccination is painless. The skin is not even broken by the needle. Sometimes a soreness develops in the armpit. If the arm becomes very sore, apply an icebag” (*4*). This advice is simple compared to the depth and breadth of information given today to a potential vaccinee. Now, volunteers are given several informational lectures and a protracted individual interview to discuss lingering questions, and they are required to sign a document confirming adequate comprehension and acceptance of the risks.

In the 1947 campaign, trouble began on April 16, when (no longer on page 1), the Times announced, “Vaccinations Stop; Drug Supply Gone; Thousands Turned Away” (*5*). With little warning, and at the height of the program, the vaccine supply vanished, something that was never explained. After spending days gearing up citizens to receive the vaccine quickly, the mayor and Dr. Weinstein now had to downplay the urgency of receiving vaccination. They assured New Yorkers that a delay of a few days or more represented “no health hazard” (*5*).

Of the 1.2 million doses distributed to date, 42,000 had been supplied by private laboratories, far short of the promised number. In contrast, the Army and Navy had given almost 800,000 doses, and the city’s public health laboratories had made another 400,000.

During the shortage, the Times noted, “hundreds of eager men, women, and children queued up at Bellevue Hospital at dawn, although vaccinations were not scheduled to begin until 10 a.m. At some stations, the crowds did not take kindly to the news that the doctors had run out of vaccine and the police had a little difficulty dispersing a crowd of several hundred” outside one vaccine station (*5*).

On April 17, the situation brightened, when more than a million doses arrived from private laboratories, and 500,000 persons were vaccinated (*6*). As the crisis slowly lessened, doctors were recruited at US$8 (US$64 in today’s market) for a 3-hour session (or US$24 for all day; US$192 in today’s market) to administer vaccine, but few volunteered. Public health authorities in Westchester County chided local physicians for charging $35 per vaccine (*7*), and a 29-year-old woman, dressed up as a nurse, vaccinated 500 people with water to impress her “man companion” until she was sent to the Bellevue psychiatric ward for evaluation (*8*).

Continued complaints about side effects were dismissed as minor nuisances by Dr. Weinstein, who again advised those whose arm ached that they only needed to place an icebag in the armpit for relief. Within a week, the program had wound down and been proclaimed “a miracle” (*2*) by all involved.

The claim of 5 or 6 million vaccinations administered cannot be reconciled against the daily tally reported in the Times. If one assumes that day-to-day numbers reported in the newspaper were roughly accurate, a simple calculation places the number of vaccinees closer to 2.5 million, far short of the announced total. On April 21, a grand total of 3.45 million recipients were reported; the next day, after noting that only 200,000 additional persons had received vaccine, the total became 4.4 million (*9*).

Although the numbers are plausible, these data reflect the difficulties intrinsic to managing such a massive program. The discrepancy may simply be a case of not adding columns of numbers in a systematic way; the fuzzy numbers do have a certain appeal to the modern, more cynical reader.

Whatever occurred, understanding the specifics of “the great vaccination miracle” of 1947 is important for maintaining equilibrium during our current smallpox vaccination program and any future programs directed at now-unanticipated infections. Not just New York City’s, but the entire country’s sense of confidence that it can handle a major rapid vaccination or pill distribution campaign leans very heavily on the apocryphal vaccine campaign of April 1947. Yet, as described above, there may be much less to the miracle than met the eye.
